# Improving Oncall Handover Through Digitalisation / a QI Project at Newham Centre for Mental Health

**DOI:** 10.1192/bjo.2022.297

**Published:** 2022-06-20

**Authors:** William Loveday, Kajanesh Ratneswaran, Georgios Nerantzis, Alexia Haysom, Nahid Hakim, Darena Dineva, Adam Richards

**Affiliations:** 1 East London NHS Foundation Trust, London, United Kingdom; 2 Essex Partnership University NHS Foundation Trust, Basildon, United Kingdom

## Abstract

**Aims:**

To improve the information exchange between oncall junior doctors and ward teams between shifts including outstanding tasks, alerts and prompts to update clinical record systems accordingly (Rio). We aimed for the handover to be circulated to the correct recipients in 95% of cases as well as to improve its content. This would minimise loss of information and improve patient safety.

**Methods:**

Handover document set up on MS Teams which is accessed by oncall junior doctors and day teams and can be updated live. Relevant training was offered to trainees during induction. We measured the number of days the document is updated and distributed and also measured the tasks not completed or not documented. We measured doctors' satisfaction via a survey.

**Results:**

We found that on average the handover document is updated and circulated correctly at a rate of 94.8% since the new MS Teams system was implemented. Participating doctors' survey showed that they felt that this system is safe and easy to use as well as reliable and more efficient than the previous system. They also noted that the training they received during induction was helpful and sufficient.

**Conclusion:**

The digitalisation of the handover process using MS Teams, developed and improved through various PDSA cycles, has resulted in a system which the users find efficient, safe and easy to use. This leads to minimisation of information losses and improves patients' safety.

